# Traits linked to sensory processing sensitivity mediate the relationship between externally oriented thinking and fantasizing

**DOI:** 10.3389/fpsyg.2024.1354120

**Published:** 2024-03-12

**Authors:** Lorna S. Jakobson, Amanda M. McQuarrie, Chantal Van Landeghem, Stephen D. Smith

**Affiliations:** ^1^Department of Psychology, University of Manitoba, Winnipeg, MB, Canada; ^2^Department of Psychology, University of Winnipeg, Winnipeg, MB, Canada

**Keywords:** alexithymia, sensory processing sensitivity, externally oriented thinking, fantasy, imagination, imagery, empathy

## Abstract

**Introduction:**

Alexithymia is characterized by difficulties identifying and describing feelings but expression of externally oriented thinking (EOT) and difficulty fantasizing is more variable. In two studies, we investigated whether links between EOT and fantasizing are mediated by sensory processing sensitivity (SPS).

**Methods:**

University students completed measures of alexithymia, SPS, and fantasizing.

**Results:**

In Study 1 (*N* = 700) we identified two clusters of SPS traits: a positive facet (sensitivity to subtle stimuli) and a negative facet (sensitivity to uncomfortable stimuli). In the 499 participants who completed the fantasy measure, low EOT scores predicted stronger SPS positive and negative traits, which predicted a stronger tendency to mentally project oneself into the lives of characters in books, movies, and plays. In Study 2 (*N* = 600), the link between EOT and this same fantasizing tendency was again mediated by features of SPS—in this case fantasy proneness and emotional reactivity.

**Discussion:**

We suggest that, whereas individuals who score high on EOT have an impoverished fantasy life, those who score relatively low on EOT and turn their attention inward are able to maintain stronger representations of imagined events in working memory (enhancing the likelihood that they will be recalled) and react more strongly to these events (enhancing their salience). Stronger expression of these features of SPS, in turn, increases the likelihood that one will develop a cognitive style that involves the application of imagery-based strategies to support deep processing of the thoughts and feelings of characters depicted in narratives.

## 1 Introduction

In 1970, Nemiah and Sifneos described a group of patients with psychosomatic illness who reported difficulties identifying their feelings and distinguishing them from bodily sensations (DIF), and difficulties describing their feelings (DDF). These patients also displayed externally oriented thinking (EOT; a strong preference to attend to external objects, people, and environmental events rather than examining their feelings) and little engagement in fantasy or imaginal activities—a cognitive style referred to as la pensée opératoire. Sifneos (1973) introduced the term *alexithymia* to refer to this constellation of features. Alexithymia is now recognized as a partially heritable trait (Karukivi and Saarijärvi, 2014) and an important transdiagnostic risk factor for a range of physical and mental health conditions (e.g., Grynberg et al., 2012).

There is strong consensus that problems with emotional appraisal (DIF/DDF) represent a core feature of alexithymia (Preece et al., 2017). However, EOT does not always accompany these problems; indeed, Jakobson and Rigby (2021) found that EOT scores can vary widely in those who meet traditional criteria for alexithymia based on their total scores on the Toronto Alexithymia Scale (TAS-20; Bagby et al., 1994). The fantasizing deficits said to be a feature of la pensée opératoire (Sifneos, 1973; Taylor et al., 2023) are also not consistently associated with scores on measures of DIF, DDF, or EOT (e.g., Haviland et al., 1991; Vorst and Bermond, 2001; Preece et al., 2017).

One possible explanation for these results is that many people who score high on alexithymia exhibit features of another personality trait that also impacts emotional awareness and emotion regulation, known as sensory processing sensitivity (SPS). For example, Rigby et al. (2020) found that almost half of the individuals who scored in the upper third of the distribution of TAS-20 total scores in their sample were classified as highly sensitive based on their scores on the Highly Sensitive Person Scale (HSPS; Aron and Aron, 1997). More recently, Van Landeghem and Jakobson (2024) found that 78.4% of individuals in their sample who scored at or above the traditional cut-off for alexithymia on the TAS-20 scored above the sample mean for total scores on the HSPS. The correlation between total scores on the TAS-20 and the HSPS is moderately strong, ranging from 0.26 to 0.39 across several studies (Rigby et al., 2020; Jakobson and Rigby, 2021; McQuarrie et al., 2023; Van Landeghem and Jakobson, 2024).

Aron et al. (2012) note that individuals with SPS are generally hypersensitive to subtle internally- or externally-generated stimuli, and to stimuli or situations they perceive to be unpleasant. Interestingly, however, they are also generally found to have “rich” inner lives, to be empathetic, and to engage in “deep” processing (Acevedo, 2020). In their classic paper, Craik and Lockhart (1972) described deep processing as encoding and processing information in a meaningful and elaborate way that supports long-term memory. Given the above, it seems plausible that individuals who score high on facets of both alexithymia and these varied features of SPS might have a different “alexithymia profile” than the patients with psychosomatic illness who were described in Nemiah and Sifneos’ (1970) classic paper. In particular, they may be less externally oriented and report stronger fantasizing. It is important to study the relationship between alexithymia and SPS in more detail as variability in the extent to which different features of these traits are expressed may have important clinical implications. We return to this point later.

The possibility that there are subtypes of alexithymia that can be distinguished, in part, on the basis of features of SPS gained support from a recent latent profile analysis (Jakobson and Rigby, 2021). In this study, five groups were identified that could be distinguished on the basis of TAS-20 subscale scores, subjective interoceptive accuracy, and sensory processing style (as measured by the Adolescent/Adult Sensory Profile; Brown and Dunn, 2002). The groups were subsequently compared on two measures of SPS: the HSPS and the Orienting Sensitivity (OS) scale from Evans and Rothbart’s (2007) Adult Temperament Questionnaire. Two of the five groups identified included a high proportion of individuals scoring in the alexithymic range on the TAS-20. Members of both groups reported marked problems with emotional appraisal (high DIF/DDF) and their HSPS profiles indicated heightened sensitivity to unpleasant stimuli or situations. However, only the group who scored low on EOT scored high on subscales of the OS that measure sensitivity to subtle stimuli. The authors argued that, if those who frequently turn their attention inward (i.e., who score low on EOT) attend more closely to mental images (which are thought to function as weak percepts; Pearson et al., 2015) this might strengthen the representations of these images in working memory (making them more salient and memorable). This could explain why, when completing the OS, the group scoring low in EOT reported being imaginative and experiencing vivid dreams (Jakobson and Rigby, 2021).

In the current paper, we tested this idea by exploring the possible mediating role that characteristics associated with SPS may play in the link between EOT and the tendency to mentally transpose oneself into the lives of characters depicted in narratives (books, movies, or plays). This tendency is often assessed using the Fantasy subscale of the Interpersonal Reactivity Index (IRI; Davis, 1980). Davis (1980) included this subscale as a measure of empathy based on earlier observations linking fantasizing tendencies to displaying heightened physiological reactivity to others and to helping behaviour (Stotland et al., 1978). This decision was supported by the subsequent finding (Davis, 1983) that scores on the Fantasy subscale correlated strongly with scores on Mehrabian and Epstein’s (1972) Emotional Empathy Scale. Not surprisingly, however, IRI Fantasy scores also correlate with measures of a range of self-oriented processes including imagination (Baron-Cohen and Wheelwright, 2004), and Bagby et al. (2020) proposed that scores on this IRI subscale might provide a reliable index of the extent to which fantasy is compromised in those with alexithymia.

Based on earlier work (e.g., Jakobson and Rigby, 2021), we predicted that individuals who were less externally oriented would tend to report stronger traits associated with SPS and that this, in turn, would predict higher IRI Fantasy scores. We tested this prediction in two studies, using data from two large, independent samples of university students. Our analyses allowed us to investigate the multidimensional nature of SPS and how its different facets relate to the features of alexithymia and to the tendency to mentally project oneself into the life of a real or fictional character.

## 2 Study 1

The first goal of Study 1 was to expand upon past research investigating the nature of SPS. In their early work in this area, Aron and Aron (1997) introduced the HSPS as a brief, unidimensional self-report measure of this trait. Subsequent studies, however, provided support for several interrelated subfactors (e.g., Meyer et al., 2005; Smolewska et al., 2006; Evans and Rothbart, 2008; Ershova et al., 2018). The three-factor model is the most widely recognized and includes a Low Sensory Threshold (LST) subfactor tapping into stimuli that make one feel uncomfortable, an Ease of Excitation (EOE) subfactor tapping into sensitivity to overstimulation, and an Aesthetic Sensitivity (AES) subfactor tapping into sensitivity to subtle, aesthetic qualities of one’s environment. However, Smolewska et al. (2006) found EOE and LST to be highly correlated and suggested that they may represent a single subfactor, which they argued was a stronger predictor of negative clinical outcomes than AES. This conclusion was supported by Liss et al. (2008) who found (a) that EOE and LST were positively associated with problems with emotional appraisal and that the combination of being easily overwhelmed and unable to identify one’s feelings was a risk marker for anxiety; and (b) that AES was conceptually distinct from EOE and LST and negatively associated with EOT. It gained further support from Jakobson and Rigby (2021) who observed that, whereas EOE and LST showed moderate positive correlations with DIF and DDF, AES was negatively correlated with EOT with moderate effect size. Attary and Ghazizadeh (2021) argued for categorizing EOE and LST as negative SPS traits closely associated with neuroticism and alexithymia (as reflected in TAS-20 total scores), and for categorizing AES as a positive SPS trait more closely related to openness—that is, to being open to new experiences, insightful, creative, and imaginative (McCrae and Cost, 1997). This suggestion is consistent with findings from a meta-analysis by Lionetti et al. (2019).

In 2012, Aron et al. noted some shortcomings of the HSPS including, for example, that it does not adequately assess sensitivity to positively valenced stimuli or the tendency to engage in deep processing. For these reasons, they recommended supplementing the HSPS with the OS scale (Evans and Rothbart, 2007). This measure includes three subscales: Neutral Perceptual Sensitivity items assess awareness of low-intensity/subtle environmental cues; Affective Perceptual Sensitivity items measure awareness of one’s emotional response to low-intensity non-social cues about one’s surroundings or conveyed through music or the visual arts; and Associative Sensitivity items assess the extent to which one engages in processes not driven by stimuli in the immediate environment, such as creative thinking, using one’s imagination, and dreaming.

Recently, De Gucht et al. (2022) developed a new 43-item measure of SPS. An exploratory factor analysis produced a general sensitivity factor and six subfactors that could be grouped into negative and positive trait clusters. Consistent with Attary and Ghazizadeh (2021), the negative cluster included two subscales tapping into the tendencies to be highly reactive/easily overwhelmed and overly sensitive to stimuli that make one feel uncomfortable, and the mean score on these two subscales was strongly correlated with scores on both the EOE and LST subscales of the HSPS (*r* ≥ 0.77). Also consistent with Attary and Ghazizadeh (2021), the positive SPS trait cluster included items assessing aesthetic sensitivity, and scores on this subscale were strongly correlated (*r* = 0.66) with scores on the AES subscale of the HSPS. In addition, the positive cluster identified by De Gucht et al. (2022) included subscales assessing sensory sensitivity to subtle internal and external stimuli and to subtle interpersonal cues, scores on which were strongly correlated with scores on the Neutral and Affective Perceptual Sensitivity subscales of the OS (0.53 ≤ *r* ≤ 0.87), and a subscale tapping into sensitivity to pleasurable forms of stimulation, scores on which were moderately correlated with AES and OS total scores (0.27 ≤ *r* ≤ 0.34). Finally, as in Attary and Ghazizadeh (2021), being highly reactive/easily overwhelmed was related to neuroticism and negative clinical outcomes, whereas aesthetic sensitivity was related to openness.

The first objective of Study 1 was to determine if the subscales of the HSPS and the OS formed negative and positive SPS clusters, as suggested by the work of Attary and Ghazizadeh (2021) and De Gucht et al. (2022). Assuming this would be the case, we also sought to examine the distribution of positive and negative SPS traits in a large sample of undergraduate students and explore the relationship between different SPS profiles and alexithymia. As suggested by past findings (Liss et al., 2008; Attary and Ghazizadeh, 2021; Jakobson and Rigby, 2021), we expected to find that high scores on DIF would be most strongly associated with high scores on the negative SPS trait cluster, and that high scores on EOT would be most strongly associated with low scores on the SPS positive trait cluster. Following this, we set out to determine whether SPS positive traits mediate the hypothesized link between EOT and impaired fantasy. We predicted that this would be the case; specifically, we predicted that individuals who turn attention inward (i.e., who have a weak external focus) would be more sensitive to subtle internally-generated stimuli (potentially leading them to experience more vivid and memorable imagery), and be more likely to mentally project themselves into scenarios depicted in narratives.

### 2.1 Materials and methods

#### 2.1.1 Participants

In Study 1 we utilized data collected (via convenience sampling) in two different research protocols. The first protocol was used by Jakobson and Rigby (2021) to collect data from a sample of 201 participants (112 women and 89 men; *M*_age_ = 19.7 years, SD = 3.9, range 17–52). Data collected by McQuarrie et al. (2023) from 305 participants who completed the second research protocol were combined with new data collected for the present investigation from 194 participants using identical procedures; this brought the final sample who completed the second protocol to 499 (385 women, 111 men, 3 non-binary or prefer not to say; *M*_age_ = 20.2 years, *SD* = 4.8; range 16 to 54). All of the 700 individuals in the total sample were students enrolled in an Introduction to Psychology course and participated to earn credit toward a research participation option. All provided informed consent prior to their participation.

#### 2.1.2 Procedures

Although both research protocols involved the completion of numerous self-report measures, participants who took part in the first protocol completed their survey at individual workstations in a computer lab that could accommodate groups of approximately 30, whereas (due to pandemic-related restrictions on in-person testing) participants who completed the second protocol completed their survey individually at a time and place of their choosing. Both protocols included items relating to demographics (age [in years] and sex [male, female, non-binary, prefer not to say]) along with measures of alexithymia and SPS. Only the second protocol included the IRI. All of the measures extracted for the present study were collected online via the Qualtrics survey platform, and descriptions of them are provided below. In addition to the above, each protocol included some measures that were not utilized in the present investigation. In particular, the first protocol included self-report measures of mental health and sensory processing style, and the second included self-report measures of depression and exposure to childhood emotional abuse. Prior to completing any self-report measures, participants in the second protocol also provided ratings of their reactions to a set of affective film clips. Descriptions of the measures not included in the present study are provided in Jakobson and Rigby (2021) and McQuarrie et al. (2023). Both protocols were approved by our university’s Research Ethics Board.

#### 2.1.3 Measures

##### 2.1.3.1 Toronto Alexithymia Scale

The 20 items comprising the TAS-20 (Bagby et al., 1994) measure three key features of alexithymia, namely DIF (7 items; e.g., *I am often confused about what emotion I am feeling*), DDF (5 items; e.g., *It is difficult for me to find the right words for my feelings*), and EOT (8 items; e.g., *Being in touch with emotions is essential* [reverse scored]). Participants indicate the extent to which they agree with each item using a 5-point Likert scale ranging from 1 = *Strongly disagree* to 5 = *Strongly agree*. Subscale scores are sums of ratings on relevant items. The TAS-20 is widely used and provides a reliable and valid measure of alexithymia (Bagby et al., 2020).

##### 2.1.3.2 Measures Assessing Sensory Processing Sensitivity

Following the recommendation of Aron et al. (2012), we used two complementary measures to capture different aspects of SPS: the 27-item HSPS (Aron and Aron, 1997) and the 15-item OS scale (Evans and Rothbart, 2007). When completing these scales, respondents indicate the extent to which each item describes them using a seven-point Likert scale. Anchors for the HSPS are 1 (*not at all*) to 7 (*extremely*) and anchors for the OS are 1 (*Extremely untrue of you*) to 7 (*Extremely true of you*). As noted earlier, the three subscales of the HSPS include EOE (12 items; e.g., *Do you find it unpleasant to have a lot going on at once?*), LST (6 items; e.g., *Are you made uncomfortable by loud noises?*) and AES (7 items; *Are you deeply moved by the arts or music?*); and the three subscales of the OS include Neutral Perceptual Sensitivity (5 items; e.g., *I often notice mild odors and fragrances*), Affective Perceptual Sensitivity (5 items; *I am often aware how the color and lighting of a room affects my mood*), and Associative Sensitivity (5 items; *When I am resting with my eyes closed, I sometimes see visual images*). Subscale scores are computed by finding the mean rating for relevant items. The HSPS possesses strong reliability and validity (Aron and Aron, 1997; Smith et al., 2019). Cronbach alphas for the OS subscales range from 0.64 to 0.79 (Evans and Rothbart, 2007).

##### 2.1.3.3 Interpersonal Reactivity Index

The IRI (Davis, 1980) includes four subscales comprised of seven items each: Fantasy (e.g., *After seeing a play or movie, I have felt as though I were one of the characters*), Perspective Taking (e.g., *I try to look at everybody’s side of a disagreement before I make a decision*), Empathic Concern (e.g., *I often have tender, concerned feelings for people less fortunate than me*), and Personal Distress (e.g., *Being in a tense emotional situation scares me*). Responses are provided on a 5-point Likert scale ranging from 0 (*Does not describe me well*) to 4 (*Describes me very well*). Subscale scores are computed by summing responses on relevant items. The subscales possess acceptable internal consistency (0.71 to 0.77) and the test–retest reliability ranges from 0.61 to 0.81 (Davis, 1980). Subscale scores relate to other measures of interpersonal functioning, emotionality, and sensitivity to others, indicating good construct validity (Davis, 1983). The original four-factor model has been recently validated by Chrysikou and Thompson (2016).

### 2.2 Results

Procedures followed when cleaning data and imputing missing values were described in our previous publications (Jakobson and Rigby, 2021; McQuarrie et al., 2023). Univariate outliers were identified and corrected through winsorizing, and linearity between independent and dependent variables was confirmed. No influential multivariate outliers were identified (Cook’s distance <0.118 for all cases). Unless otherwise indicated, statistical analyses in both studies were conducted using IBM SPSS Statistics for Windows (v 28), and an alpha of 0.05 was adopted for tests of statistical significance.

Using G*Power 3.1 (Faul et al., 2009) we determined that the sample size provided ample power (>0.80) for a planned analysis of variance (ANOVA) assuming a medium effect size. Based on the standard rule-of-thumb of 10 observations per parameter and on guidelines put forth by Watkins (2021), the sizes of the two subsamples also provided sufficient power for the planned mediation and factor analyses, respectively.

#### 2.2.1 How do the HSPS and OS subscales relate to one another?

An exploratory factor analysis was completed (using R Statistical Software, version 4.3.1) to determine the underlying factor structure of the subscales of the HSPS and OS. This analysis was conducted with data from the 201 participants who completed the first research protocol (the calibration sample). First, the appropriateness of conducting the analysis was confirmed by identifying the correlations between variables, ruling out multicollinearity, and calculating both Bartlett’s (1950) test of sphericity and the Kaiser-Meyer-Olkin (KMO) measure of sampling adequacy (Kaiser, 1974). Based on the recommendations of Velicer et al. (2000), both parallel analysis (PA; Horn, 1965) and minimum average partial (MAP; Velicer, 1976) extraction methods were used to determine the number of factors to retain for rotation. Factor rotation was completed utilizing an oblique rotation method, given the fact that the variables were correlated. Factor loadings less than 0.32 were rejected as not meaningful (Watkins, 2021). Finally, factor scores were calculated utilizing the regression method.

Results from the PA and MAP provided support for one or two factors. The one factor model produced a root mean squared residual (RMSR) value of 0.117, which is above the recommended cutoff of ≤0.08 (Brown, 2015). Additionally, over half of the individual residual correlations were greater than 0.05. This indicates that the one factor model did not extract enough factors (Watkins, 2021). The two-factor model converged properly and produced salient loadings onto each factor. It produced a RMSR value of 0.019 and all residual correlations were less than 0.05. As seen in Table 1, the EOE and LST subscales loaded onto Factor 1, and the AES and the three OS subscales loaded onto Factor 2. Following Attary and Ghazizadeh (2021) and De Gucht et al. (2022), factor 1 was characterized as capturing a negative SPS trait cluster and factor 2 a positive SPS trait cluster.

**Table 1:** Factor loadings for the HSPS and OS subscales in the exploratory factor analysis.

Subscale	Factor 1	Factor 2
	Negative SPS trait cluster	Positive SPS trait cluster
EOE	**0.920**	−0.122
LST	**0.624**	−0.090
APS	0.152	**0.690**
AS	0.045	**0.638**
NPS	−0.156	**0.351**
AES	0.277	**0.681**

A cut off factor loading score of 0.32 was used; scores above this are shown in bold font. Subscales of the Highly Sensitive Person Scale (HSPS): EOE, Ease of Excitation; LST, Low Sensory Threshold; AES, Aesthetic Sensitivity. Subscales of the Orienting Sensitivity (OS) scale: APS, Affective Perceptual Sensitivity; NPS, Neutral Perceptual Sensitivity; AS, Associative Sensitivity.

**Figure 1:**
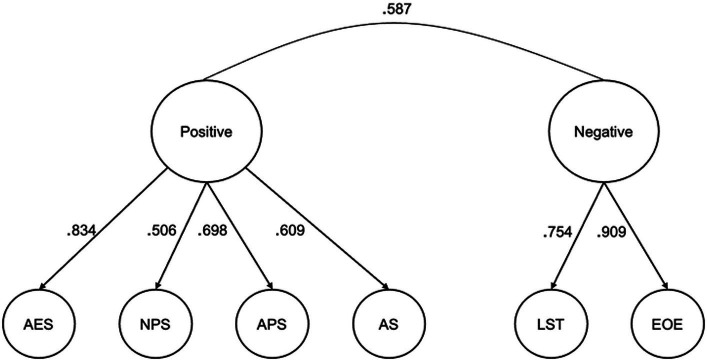
Standardized loading estimates for the positive and negative aspects of SPS in the two-factor model identified using confirmatory factor analysis. Subscales of the Highly Sensitive Person Scale (HSPS): EOE, Ease of Excitation; LST, Low Sensory Threshold; AES, Aesthetic Sensitivity. Subscales of the Orienting Sensitivity (OS) scale of the Adult Temperament Questionnaire (short form): APS, Affective Perceptual Sensitivity; NPS, Neutral Perceptual Sensitivity; AS, Associative Sensitivity.

Next, the one- and two-factor models were evaluated with confirmatory factor analysis using data from the 499 participants who completed the second research protocol (the validation sample). Little’s Missing Completely at Random test confirmed that missing data were missing completely at random. Missing values were then imputed using an Estimation-Maximization algorithm. As the data met the assumption of multivariate normality, the maximum likelihood estimation extraction method was used. Although chi-square test results are often considered when assessing fit, this test is sensitive to sample size, leading to failure to reject poorly fitting models when sample size is small (< 200) and to rejection of adequate models when sample size is large (Hu et al., 1992; Gatignon, 2010; Singh et al., 2016). For this reason, we focused on three other indicators when assessing goodness-of-fit for the two tested models: the comparative fit index (CFI), the Tucker-Lewis Index (TLI), and the root mean square error of approximation (RMSEA). A CFI value of 0.95 indicates good fit (Garson, 2023), a TLI value of 0.90 (Byrne, 1994) or 0.95 (Garson, 2023) indicates good fit, and a RMSEA value of 0.05 indicates good fit although values from 0.08 to 0.10 are taken as evidence of mediocre fit (Browne and Cudeck, 1993; Garson, 2023). Table 2 presents the fit indices for the two tested models. The one-factor model (in which each of the six subscales load onto an overarching “SPS” factor) produced CFI and TLI values below and RMSEA values above the recommended cutoffs, suggesting that it did not provide a good fit for the data. The two-factor model (which included positive and negative SPS trait clusters) produced good fit based on CFI and TLI values and mediocre fit based on the RMSEA value; overall, then, the fit of the two-factor model was deemed to be acceptable. The standardized loading estimates for the two-factor model are shown in Figure 1.

**Table 2:** Confirmatory factor analysis fit indices.

	*Χ^2^*	df	CFI	TLI	RMSEA [90% CI]
One-factor model	225.088*	9	0.774	0.624	0.219 [0.195, 0.245]
Two-factor model	33.596*	8	0.973	0.95	0.080 [0.053, 0.109]

df, degrees of freedom; CFI, comparative fit index; TLI, Tucker-Lewis fit index; RMSEA, root mean square error of approximation; CI, confidence interval.

*p < 0.01.

In both the calibration and the validation sample, the negative SPS factor score was almost perfectly correlated with the average score on the EOE and LST subscales and the positive SPS factor score was almost perfectly correlated with the average score on the AES and the three OS subscales (*r* > 0.960 in all cases). Given this, in the analyses described below we used these composite scores to quantify the strength of SPS negative and SPS positive traits, respectively.

#### 2.2.2 What is the distribution of positive and negative SPS traits and what is their relationship to alexithymia?

Descriptive statistics for and zero-order correlations between the study variables are shown in Table 3 (see Supplementary Tables S1, S2 for results for males and females, separately).

**Table 3:** Descriptive statistics and zero-order correlations between study 1 variables.

Variable	*M*	*SD*	2-tailed Pearson correlations^a^							
			df = 700				df = 499			
			DIF	EOT	SPS pos	SPS neg	EC	FS	PD	PT
DDF	15.1	4.8	0.664	0.277	−0.001	0.243	−0.080	−0.035	0.318	−0.139
DIF	17.8	6.4	–	0.184	0.127	0.400	0.025	0.046	0.407	−0.119
EOT	19.3	4.2		–	−0.445	−0.055	−0.254	−0.224	0.181	−0.313
SPS pos	4.7	0.8			–	0.386	0.359	0.406	0.061	0.366
SPS neg	4.0	1.1				–	0.273	0.240	0.512	0.125
EC	2.9	0.7					–	0.340	0.228	0.428
FS	2.6	0.8						–	0.080	0.185
PD	1.8	0.7							–	−0.017
PT	2.5	0.7								–

Subscales of the Toronto Alexithymia Scale (TAS-20): DDF, difficulty describing feelings; DIF, difficulty identifying feelings; EOT, externally oriented thinking. SPS pos and SPS neg are the positive and negative composite scores for traits associated with sensory processing sensitivity. Subscales of the Interpersonal Reactivity Index (IRI): EC, Empathic concern; FS, Fantasy; PD, Personal distress; PT, Perspective Taking.

a Values shown in gold, orange, and red represent small, medium, and large effect sizes, respectively; all are significant at the *p* ≤ 0.008 level.

**Figure 2:**
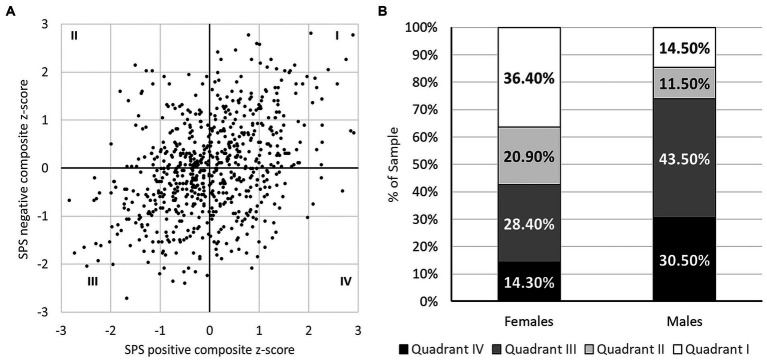
Distribution of positive and negative traits associated with sensory processing sensitivity (SPS) in the full sample **(A)** and in males and females **(B)**. The four SPS groups include individuals who: scored high on both positive and negative SPS traits (quadrant I), high on negative traits only (quadrant II), low on both trait clusters (quadrant III), and high on positive traits only (quadrant IV).

As expected, the negative and positive SPS composite scores were positively correlated with one another, *r*(700) = 0.386, *p* < 0.001. However, as can be clearly seen in Figure 2A, any combination of scores was possible. Thus, individuals could score high on one trait cluster but low on the other (quadrants II and IV), although the majority scored either high on both (quadrant I) or low on both (quadrant III). The number of individuals whose scores fell in each quadrant were as follows: quadrant I (high positive, high negative) = 213, quadrant II (low positive, high negative) = 127, quadrant III (low positive, low negative) = 228, quadrant IV (high positive, low negative) = 132.

A chi-square test compared the SPS profiles of males and females. (Note that this analysis excluded the three individuals who identified as non-binary or did not disclose their sex.) As shown in Figure 2B, the proportion of females in quadrants I and II was higher than the proportion of males in corresponding quadrants, whereas the reverse was true in quadrants III and IV, *Χ*^2^(3) = 59.47, *p* < 0.001. Two-sided independent samples *t*-tests confirmed that although females scored higher than males on both positive SPS traits [*M*_female_ = 4.75, *SD* 0.78, *M*_male_ = 4.61, *SD* 0.75, *t*(695) = 2.27, *p* = 0.004, *d* = 0.19] and negative SPS traits [*M*_female_ = 4.19, *SD* 1.06, *M*_male_ = 3.52, *SD* 0.89, *t*(695) = 7.88, *p* = 0.023, *d* = 0.66], the latter effect was considerably larger. Follow-up ANOVAs that included both sex and the protocol completed as grouping variables confirmed that mean composite scores were similar and sex differences were as described above regardless of which protocol had been completed.

**Figure 3:**
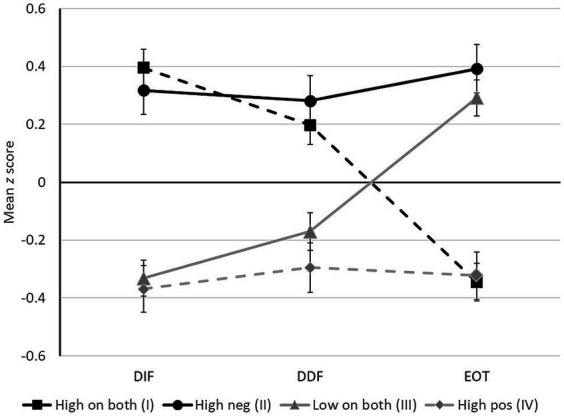
Comparison of subscale scores on the Toronto Alexithymia Scale across groups with different sensory processing sensitivity (SPS) profiles. The figure shows mean *z* scores (*SE* indicated) on three subscales: DIF, difficulty identifying feelings; DDF, difficulty describing feelings; EOT, externally oriented thinking. The four SPS groups include individuals who: scored high on both positive and negative SPS traits (quadrant I), high on negative traits only (quadrant II), low on both trait clusters (quadrant III), and high on positive traits only (quadrant IV).

Individuals whose scores fell in quadrants I and II (who reported strong negative SPS traits) were at highest risk for alexithymia, with 61.4 and 69.3% having TAS-20 total scores that fell in the borderline-to-alexithymic range (≥ 52), respectively. In a follow-up analysis, we compared the TAS-20 profiles of those whose SPS composite scores fell in different quadrants using a mixed ANOVA, with Greenhouse–Geisser adjustment to the degrees of freedom where indicated. DIF, DDF, and EOT scores were converted to *z* scores in this analysis to put them on a common scale. A significant Quadrant X Subscale interaction [*F*(5.2, 1195.6) = 33.92, *p* < 0.001, *η_p_*^2^ = 0.128; see Figure 3] was followed up with tests of simple main effects, which revealed two key findings. First, DDF and DIF scores were higher in quadrants I and II than in quadrants III and IV (all contrasts *p* ≤ 0.001), suggesting that problems with emotional appraisal are characteristic of those reporting strong negative SPS traits. Second, EOT scores were higher in quadrants II and III than in quadrants I and IV (all contrasts *p* ≤ 0.001), supporting the view that being externally oriented (i.e., failing to turn attention inward) is characteristic of those reporting weak positive SPS traits. The net effect was that the two groups at highest risk for alexithymia (quadrants I and II) could be distinguished primarily on the basis of their EOT scores. The same was true of the two groups at lowest risk for alexithymia (quadrants III and IV). When the research protocol that had been completed was added as an additional grouping variable there was no main effect of protocol and no interactions involving protocol, with the Quadrant X Subscale interaction taking the same form in both samples.

#### 2.2.3 Does SPS mediate the link between EOT and IRI fantasy scores?

The mediation analysis was conducted using data collected as part of the second research protocol (*n* = 499), which was the only one that included the IRI. Before testing for mediation, we examined the zero-order Pearson correlations between our personality measures and the four subscales of the IRI (see Table 3). EOT showed a moderately strong negative relationship not only to scores on the IRI Fantasy scale (as predicted), but on the Empathic Concern and Perspective Taking subscales as well. Scores on these three IRI subscales were also found to be moderately positively correlated with both clusters of SPS traits. In contrast, DDF and DIF showed the strongest relationships to the Personal Distress subscale of the IRI, which assesses the self-oriented tendency to feel anxious and uneasy in emotionally charged situations. Scores on this subscale were strongly related to negative SPS traits. (Note: *p* ≤ 0.005 for all correlations reported above.)

To test whether positive and/or negative SPS scores mediated the link between EOT and IRI Fantasy scores we ran a mediation analysis using Hayes’ PROCESS model 4. DIF and DDF scores were included as covariates. In this analysis and a subsequent mediation (see Study 2), the significance of the indirect effect was determined by examination of percentile bootstrap confidence intervals (BCIs) based on 5,000 bootstrap samples. Full mediation via both SPS positive traits (indirect effect = −0.030, Boot SE = 0.005, BCI [−0.041, −0.020]) and SPS negative traits (indirect effect = −0.003, Boot SE = 0.002, BCI [−0.006, −0.0001]) was supported (Table 4, Model A), although the indirect effect through SPS positive traits was significantly larger (indirect effect contrast = 0.027, Boot SE 0.006 [0.016, 0.040]). As predicted, after controlling for problems with emotional appraisal, being better able to direct attention inward (low EOT) predicted greater sensory sensitivity (particularly to subtle stimuli) and this, in turn, predicted higher IRI Fantasy scores. Importantly, the same result was obtained when we reran the mediation after excluding from the calculation of the SPS positive score the items from the Associative Sensitivity subscale of the OS scale, which directly addresses engagement in internal processes such as dreaming and imagery (see Table 4, Model B). This suggests that the link between SPS positive traits and IRI Fantasy scores was not due to item overlap but instead reflects the fact that heightened sensitivity to subtle stimuli is common in those reporting strong fantasizing.

**Table 4:** Unstandardized effects in tests for mediation of the link between EOT and fantasy scores.

	Outcome	Predictors	Coefficient	*SE*	LLCI	ULCI	*R^2^*	*F*
Model A^a^	SPS neg	Constant	3.510	0.217	3.084	3.936	0.188	38.08***
		**EOT**	**−0.033**	**0.011**	**−0.054**	**−0.011**		df (3, 495)
		DDF	−0.009	0.013	−0.034	0.017		
		**DIF**	**0.076**	**0.009**	**0.058**	**0.094**		
	SPS pos^a^	Constant	6.102	0.154	5.798	6.405	0.259	57.76***
		**EOT**	**−0.094**	**0.008**	**−0.109**	**−0.079**		df (3, 495)
		DDF	−0.007	0.009	−0.025	0.011		
		**DIF**	**0.030**	**0.007**	**0.018**	**0.043**		
	IRI fantasy	Constant	1.019	0.314	0.403	1.636	0.175	20.98***
		EOT	−0.010	0.009	−0.027	0.008		df (5, 493)
		**SPS neg**	**0.077**	**0.035**	**0.009**	**0.145**		
		**SPS pos**	**0.317**	**0.048**	**0.222**	**0.412**		
		DDF	−0.004	0.009	−0.022	0.014		
		DIF	−0.001	0.007	−0.015	0.013		
Model B^a^	SPS neg	Constant	3.510	0.000	3.084	3.936	0.188	38.08***
		**EOT**	**−0.033**	**0.003**	**−0.054**	**−0.011**		df (3, 495)
		DDF	−0.009	0.507	−0.034	0.017		
		**DIF**	**0.076**	**0.000**	**0.058**	**0.094**		
	SPS pos	Constant	6.127	0.000	5.821	6.432	0.255	56.42***
		**EOT**	**−0.094**	**0.000**	**−0.109**	**−0.079**		df (3, 495)
		DDF	−0.010	0.268	−0.028	0.008		
		**DIF**	**0.029**	**0.000**	**0.016**	**0.042**		
	IRI fantasy	Constant	1.183	0.000	0.563	1.802	0.162	19.10***
		EOT	−0.012	0.164	−0.030	0.005		df (5, 493)
		**SPS neg**	**0.081**	**0.022**	**0.012**	**0.149**		
		**SPS pos**	**0.287**	**0.000**	**0.191**	**0.383**		
		DDF	−0.003	0.709	−0.021	0.015		
		DIF	0.000	0.980	−0.014	0.014		

Subscales of the Toronto Alexithymia Scale: EOT, externally oriented thinking; DIF, difficulty identifying feelings; DDF, difficulty describing feelings. SPS neg and SPS pos, negative and positive facets of sensory processing sensitivity, respectively. LLCI and ULCI, lower limit and upper limit of the 95% confidence interval. Bolded coefficients are significant.

a Model A includes the Associative Sensitivity subscale in the SPS pos composite score, whereas Model B does not.

***p < 0.001.

**Figure 4:**
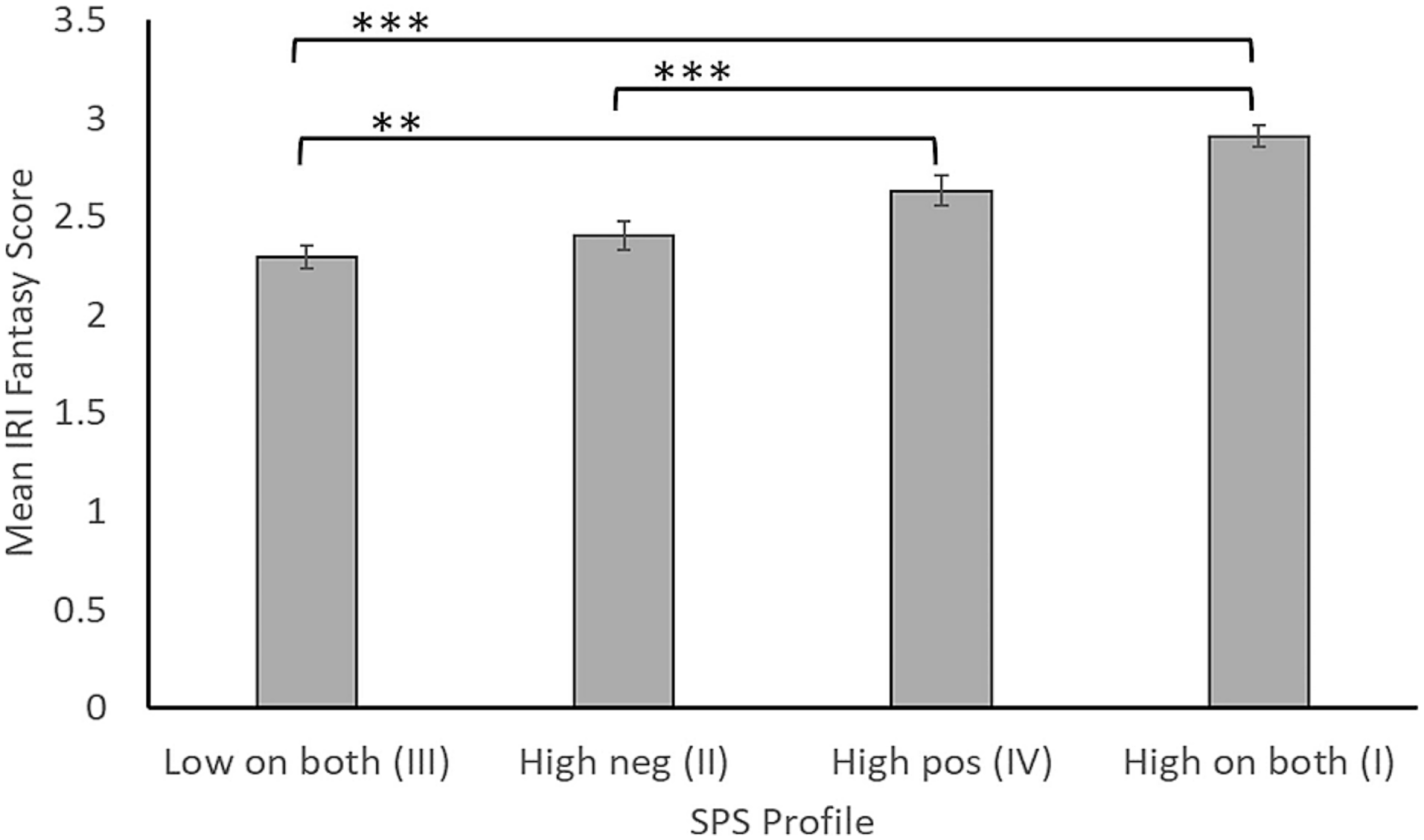
Mean scores on the Fantasy subscale of the Interpersonal Reactivity Index (SE indicated) for four groups displaying different levels of positive and negative traits linked to sensory processing sensitivity (SPS): those who scored high on both positive and negative SPS traits (quadrant I), high on negative traits only (quadrant II), low on both trait clusters (quadrant III) and high on positive traits only (quadrant IV). ** *p* < 0.01; *** *p* < 0.001.

It is important to remember that people vary in their SPS profiles (see above). To explore how IRI Fantasy scores varied as a function of one’s SPS profile, we used a between-subjects ANOVA to compare IRI Fantasy scores of those whose SPS composite *z* scores had fallen in quadrant I (high on both positive and negative SPS traits, *n* = 162), quadrant II (high on negative SPS traits only, *n* = 89), quadrant III (low on both trait clusters, *n* = 159), and quadrant IV (high on positive SPS traits only, *n* = 89) of Figure 2A. The main effect of group was significant, *F*(3, 495) = 21.89, *p* < 0.001, *η_p_*^2^ = 0.117 (see Figure 4). Post-hoc tests (with Bonferroni correction to the alpha level) confirmed that, regardless of where they scored on SPS negative traits, individuals who scored high on SPS positive traits had higher Fantasy scores than the group who scored low on both traits. These findings lend additional support to the view that SPS positive traits are the best overall predictor of the tendency to imaginatively transpose oneself into the lives of fictional or real characters.

### 2.3 Discussion

Study 1 produced several noteworthy findings. First, consistent with other reports (Attary and Ghazizadeh, 2021; De Gucht et al., 2022), we found support for negative and positive clusters of SPS traits. The fact that the negative traits (which reflect sensitivity to aspects of the sensory environment that make one uncomfortable) were more strongly related to the tendency to feel discomfort during tense interpersonal situations (as indexed by high IRI Personal Distress scores) is consistent with the idea that this trait cluster is associated with high levels of neuroticism (Attary and Ghazizadeh, 2021; De Gucht et al., 2022). Although both clusters of SPS traits correlated positively with scores on the remaining IRI subscales, it is possible that the higher levels of personal distress experienced by those exhibiting strong negative SPS traits might interfere with their ability to appraise and regulate negative emotions, making it difficult for them to act with compassion in real-life situations (see Jordan et al., 2016). Future studies that incorporate objective tests of prosocial action tendencies are needed to determine if this is the case.

A second noteworthy finding was that, consistent with De Gucht et al. (2022) and Konrad and Herzberg (2019), females scored higher than males on both clusters of SPS traits, with the group difference being particularly large for the negative cluster. As the negative SPS trait cluster has been linked in past work to neuroticism and negative clinical outcomes (Attary and Ghazizadeh, 2021; De Gucht et al., 2022), these findings may help to explain (in part) why females are routinely found to score higher than males on measures of stress-related psychopathology (e.g., Bangasser et al., 2010).

A third key finding from this study was that different SPS profiles were linked to distinctly different patterns of TAS-20 subscale scores. The patterns we observed after grouping participants on the basis of their SPS profiles mirror those described in the subset (28.7%) of the current sample who took part in the study by Jakobson and Rigby (2021). Thus, in that study we identified: (a) two groups at high risk for alexithymia who scored high on DIF/DDF and on SPS negative traits (like those in quadrants I and II of the present study); (b) two groups who scored relatively low on EOT and above-average on SPS positive traits (like those in quadrants I and IV of the present study); and (c) one group who scored low on DIF/DDF but relatively high on EOT and reported few symptoms of SPS (like those in quadrant III of the present study). Note that only participants who reported strong positive but weak negative SPS traits (quadrant IV) had uniformly low TAS-20 subscale scores; they would generally be classified as “lexithymic” on these grounds. Given that poor mental health outcomes have been more strongly associated with negative than positive SPS traits (e.g., De Gucht et al., 2022), one might predict that these “lexithymic” individuals might include a number of highly sensitive people who—while still being moderately anxious—would be better able to cope with feelings of discomfort and sensory overload than those who score higher on negative than positive SPS traits. Although not tested here, it is possible that reporting stronger positive than negative SPS traits is a feature of highly sensitive individuals who were raised in supportive environments. Such people have been found to be better able to understand and manage their emotions than highly sensitive people who experienced early adversity (Aron et al., 2012; Greven et al., 2019). This may reflect the fact that these kinds of experiences are associated with higher levels of alexithymia (Khan and Jaffee, 2022). Interestingly, Karaca Dinç et al. (2021) found that both SPS and alexithymia mediated the relationship between childhood trauma and psychopathology, including depression, anxiety, and negative self-esteem.

The fourth key finding from Study 1 was that SPS positive traits and, to a lesser extent, SPS negative traits, mediated the link between EOT and IRI Fantasy scores, when variance related to problems with emotional appraisal were held constant. This finding may help resolve past arguments about the nature of alexithymia. We propose that whereas problems with emotional appraisal (DIF/DDF) are core features of alexithymia, there is variability in the extent to which these difficulties are accompanied by EOT and deficits in fantasy. Alexithymic individuals who exhibit these latter two features are relatively insensitive to subtle, internally- and externally-generated stimuli, including mental images. In contrast, alexithymic individuals who turn their attention inward tend to be highly sensitive to such stimuli and report strong engagement in fantasizing. Although, as suggested above, members of both groups would be at elevated risk for mental health problems, especially if they had been exposed to early adversity (Aron et al., 2012), findings from Jakobson and Rigby (2021) suggest that this risk would be particularly high in members of the latter group whose extreme sensitivity would exacerbate problems with emotion regulation.

## 3 Study 2

In Study 1, the IRI Fantasy scale served as our measure of the extent to which fantasy is compromised in those with alexithymia. However, as noted earlier, this measure assesses more than simply having a rich inner life. Murphy et al. (2020) argue that it also captures a characteristic closely related to Tellegen’s absorption construct (Tellegen and Atkinson, 1974); indeed, these two measures are highly correlated (Wickramasekera and Szlyk, 2003). Tellegen and Atkinson (1974) define absorption as a disposition to fully engage one’s attention in sensory and imaginative experiences in ways that alter one’s perception, memory, and mood. As such, one might argue that what the IRI Fantasy scale really captures is a high-level feature of SPS, namely strong depth of processing (Aron et al., 2012). People who process information deeply often generate mental images or visual representations that they connect to prior knowledge in working memory when evaluating, analyzing, and synthesizing information, leading to better recall (Craik and Lockhart, 1972). By imagining themselves in circumstances like those facing characters (real or fictional) and drawing on their own past experiences, individuals who deeply process narratives can gain a better understanding of the characters’ thoughts and feelings. In Study 2, we tested the possibility that the link between EOT and the adoption of this type of imagery-based processing style might be mediated by two features of SPS: emotional reactivity and fantasy proneness.

In addition to the IRI, we administered the Bermond-Vorst Alexithymia Questionnaire (BVAQ; Vorst and Bermond, 2001). The authors contend that the characteristics the BVAQ samples can be grouped into two clusters. The Identifying, Verbalizing, and Analyzing subscales measure what the authors refer to as “cognitive” alexithymic traits; they correspond to the DIF, DDF, and EOT subscales of the TAS-20, respectively, and total scores in the cognitive domain are strongly correlated with TAS-20 total scores (Vorst and Bermond, 2001). The remaining two subscales (Emotionalizing and Fantasizing) measure what the authors of the BVAQ refer to as “affective” alexithymic traits, with higher scores reflecting lower levels of emotional reactivity and fantasy proneness. We would point out, however, that *low* scores on these two subscales could alternatively be viewed as indexing traits characteristic of those with SPS. Conceptualizing low scores in this (unconventional) way may clarify why the Type I and Type II alexithymia subtypes identified by Moormann et al. (2008) bear some similarities to the two alexithymic subtypes identified by Jakobson and Rigby (2021) that were described earlier (although, as will be discussed later, differences are also apparent). Thus, Moormann et al. (2008) describe individuals with both Type I and Type II alexithymia as having poorly developed cognitions regarding emotions, but state that those with a Type II profile are more emotionally reactive and imaginative (in other words, whereas those with a Type I profile score high on both the cognitive and affective domains, those with a Type II profile only score high on the former). Interestingly, as we might expect based on the results of Study 1, in the English version of the BVAQ (which was used in the current study) Emotionalizing and Fantasizing scores are positively correlated with scores on Analyzing, with moderate effect size (Vorst and Bermond, 2001); thus, those who are more prone to look inward tend to report being more emotionally reactive and fantasy prone.

Items in the Emotionalizing subscale have good face validity as measures of the emotional reactivity seen in SPS. They address the extent to which an individual becomes emotionally aroused by certain kinds of events. Half of the items refer to events that are negatively valenced (e.g., someone else crying uncontrollably), but the remaining items refer to events that are positively valenced (e.g., being around people who are wildly enthusiastic about something) or simply unexpected. People with SPS report being emotionally reactive (Aron et al., 2012), particularly in response to negative events (Van Reyn et al., 2023). In objective testing they show stronger behavioural (Jagiellowicz et al., 2016) and neural responses (Acevedo et al., 2014) to both positively and negatively valenced stimuli compared to those who are less sensitive. Aron et al. (2012) argue that heightened emotional reactivity amplifies the salience of events, which promotes deeper cognitive processing of them.

Items in the Fantasizing subscale of the BVAQ have good face validity as measures of fantasy proneness—specifically, the frequency with which one daydreams, fantasizes, or uses their imagination, and the pleasure one gets from doing so. Many people scoring high on SPS engage in these activities frequently (e.g., Bröhl et al., 2022), describing themselves as having rich inner lives (Aron and Aron, 1997). This “richness” extends to involuntary forms of imagination such as dreams; thus, those scoring high on SPS report frequent dreams (Schredl et al., 2020) along with intensely positive dreams and frequent nightmares (Carr and Nielsen, 2017), suggesting that their heightened sensitivity influences processing during sleep (Carr et al., 2021). Schredl et al. (2022) linked aesthetic sensitivity and being sensitive to stimuli that make one uncomfortable (LST) to lucid dream frequency, and Carr and colleagues (Carr and Nielsen, 2017; Carr et al., 2020, 2021) found that the link between SPS and nightmare frequency was mediated by nightmare distress and emotional reactivity to adverse environments and moods. Together, these findings suggest that both positive and negative features of SPS may influence dream recall, and that the negative factors may be important in amplifying our reactions to negative dream content. Interestingly, Levin and Fireman (2001) linked nightmare recall frequency to both fantasy proneness and absorption, and Khodarahim et al. (2023) showed that SPS moderated the link between negative affectivity and a latent variable reflecting both dream recall frequency and attitudes toward dreams. Specifically, negative affectivity predicted SPS, which predicted more frequent dream recall and a stronger tendency to regard dreams as meaningful—the latter variable possibly reflecting deeper processing of dream content.

Given the above, in Study 2 we addressed the question of whether emotional reactivity and fantasy proneness (as measured by the BVAQ) would mediate the link between EOT (captured by the Analyzing subscale) and IRI Fantasy scores. To do this, we performed a secondary analysis of data collected in a previously published report that included a large, undergraduate sample (Van Landeghem et al., 2019). We predicted that individuals who turn attention inward (i.e., who have a weak external focus) would maintain stronger representations of imagined events in working memory (increasing awareness of how frequently they occur) and react more strongly to these events (further enhancing their salience), and that both of these effects would promote the development of a cognitive style involving the use of imagery-based strategies to support deep processing of narratives (including the thoughts and feelings of the characters depicted), as reflected in high IRI Fantasy scores.

### 3.1 Materials and methods

#### 3.1.1 Participants

Study 2 included a convenience sample of 600 participants (382 women, 199 men, 19 sex not disclosed; *M*_age_ = 18.9 years, *SD* = 2.9). Participants were students enrolled in an Introduction to Psychology course who participated to earn credit toward a research participation option. They provided informed consent prior to their participation.

#### 3.1.2 Procedures and measures

Study 2 included numerous self-report measures, which were completed in two phases. Participants first supplied demographic information and completed the IRI (Davis, 1980) (presented in paper form) as part of a pre-screening survey administered in-class to assist investigators in identifying candidates for their studies. Anyone who had completed the IRI in phase one was invited to volunteer for our study. In phase two, eligible participants who consented to do so went on to complete measures assessing exercise dependence, disordered eating, alexithymia, and depression in that order. These data were collected via the Qualtrics survey platform, at a time and place of participants’ choosing. Only data from the IRI and the alexithymia measure (the BVAQ) were used in the present study. Descriptions of the remaining measures are available in Van Landeghem et al. (2019). The study protocol was approved by our university’s Research Ethics Board.

#### 3.1.3 Interpersonal Reactivity Index

For a description of the IRI see Study 1. Note, however, that in the pre-screening survey mentioned above the items comprising the IRI were responded to on a 10-point scale rather than the standard 5-point scale; the anchors were 1 = *Does not describe me well* to 10 = *Describes me very well*. Cronbach alphas for all subscales were ≥ 0.728 in the current sample.

#### 3.1.4 Bermond-Vorst Alexithymia Questionnaire

The BVAQ is a 40-item measure that includes five 8-item subscales: Identifying Emotions (e.g., *When I am tense, it remains unclear from which of my feelings this comes*); Verbalizing Emotions (e.g., *I find it difficult to express my feelings verbally*); Analyzing Emotions (e.g., *I hardly ever consider my feelings*); Emotionalizing (e.g., *Unexpected events often overwhelm me with emotion* [reverse scored]); and Fantasizing (e.g., *I often use my imagination* [reverse scored]). Participants respond to each item using a 5-point Likert scale ranging from 1 (*This definitely applies*) to 5 (*This in no way applies*). After reverse-scoring half the items, subscale scores are extracted by summing relevant items. High scores on the Identifying, Describing, and Analyzing subscales are indicative of experiencing stronger DIF, DDF, and EOT, respectively; in contrast, low scores on the Emotionalizing and Fantasizing subscales suggest stronger emotional reactivity and more frequent fantasizing. Previous studies support the five-factor structure and psychometric properties of the BVAQ (Berthoz et al., 2000; Vorst and Bermond, 2001). Cronbach alphas for the five subscales of the BVAQ were ≥ 0.711 in the current sample.

### 3.2 Results

Procedures followed when cleaning and imputing missing data were described in Van Landeghem et al. (2019). Univariate outliers were identified and corrected through winsorizing, and linearity between independent and dependent variables was confirmed. No influential multivariate outliers were identified (Cook’s distance <0.211 for all cases). Based on the standard rule-of-thumb of 10 observations per parameter the sample size provided ample power for the planned mediation analysis.

Descriptive statistics for and zero-order correlations between the study variables are shown in Table 5 (see Supplementary Tables S3, S4 for results for males and females, separately).

**Table 5:** Descriptive statistics and intercorrelations between study 2 variables (*N* = 600).

Variable	*M*	*SD*	2-tailed Pearson correlations^a^							
			BVAQ I	BVAQ A	BVAQ E	BVAQ F	EC	FS	PD	PT
BVAQ V	24.4	7.5	0.379	0.448	0.070	−0.019	−0.093	−0.089	0.118	−0.128
BVAQ I	20.1	5.7	–	0.350	−0.106	0.036	−0.006	0.029	0.261	−0.160
BVAQ A	18.4	4.9		–	0.410	0.220	−0.225	−0.212	−0.016	−0.228
BVAQ E	20.7	5.5			–	0.161	−0.444	−0.310	−0.449	−0.041
BVAQ F	18.9	5.9				–	−0.048	−0.397	−0.074	−0.099
EC^b^	50.5	9.7					–	0.308	0.267	0.395
FS^b^	43.5	11.5						–	0.228	0.137
PD^b^	34.7	9.7							–	−0.106
PT^b^	45.4	10.0								–

Subscales of the Bermond-Vorst Alexithymia Questionnaire (BVAQ): V, Verbalizing; I, Identifying; A, Analyzing; E, Emotionalizing; F, Fantasizing. Subscales of the Interpersonal Reactivity Index (IRI): EC, Empathic concern; FS, Fantasy; PD, Personal Distress; PT, Pesrspective Taking.

a Values shown in gold and orange represent small and medium effect sizes, respectively; all are significant at the *p* ≤ 0.009 level.

b Items on the IRI were rated on a Likert scale ranging from 1 to 10, rather than the usual 1 to 5.

We first examined the zero-order Pearson correlations between our personality measures and the four subscales of the IRI (see Table 5). As in Study 1, having an external focus (high Analyzing) was negatively related to scores on the IRI Fantasy, Empathic Concern, and Perspective Taking subscales, and problems with emotional appraisal (high Verbalizing and Identifying) were positively related to IRI Personal Distress scores. Regarding the two putative measures of SPS, we observed that whereas both fantasy proneness (low Fantasizing) and being more emotionally reactive (low Emotionalizing) predicted higher scores on the IRI Fantasy scale (as expected), only reporting greater emotional reactivity predicted higher scores on Empathic Concern and Personal Distress.

To test whether emotional reactivity and/or fantasy proneness mediated the link between Analyzing and IRI Fantasy scores, we ran a mediation analysis using Hayes’ PROCESS model 4. Identifying and Verbalizing scores were included as covariates in this analysis. Full mediation was supported via both Emotionalizing (indirect effect −0.289, Boot SE 0.056, BCI [−0.401, −0.185]) and Fantasizing (indirect effect −0.242, Boot SE 0.048, BCI [−0.343, −0.155]) (see Table 6). The indirect effects were of similar magnitude (indirect effect contrast = −0.047, Boot SE 0.074 [−0.188, 0.097]). After controlling for problems with emotional appraisal, being better able to direct attention inward (low Analyzing) predicted being more reactive (low Emotionalizing) and engaging in more frequent fantasies (low Fantasizing), both of which predicted higher IRI Fantasy scores. In addition to the above, experiencing more problems describing one’s emotions (high Verbalizing) predicted both greater fantasy proneness (low Fantasizing) and lower IRI Fantasy scores when controlling for all other variables.

**Table 6:** Unstandardized effects in tests for mediation of the link between analyzing and IRI fantasy scores.

Outcome	Predictors	Coefficient	SE	LLCI	ULCI	*R^2^*	*F*
Emotionalizing	Constant	16.144	0.924	14.329	17.959	0.243	63.59***
	**Analyzing**	**0.598**	**0.046**	**0.508**	**0.687**		df (3, 596)
	Verbalizing	−0.050	0.031	−0.110	0.010		
	**Identifying**	**−0.260**	**0.038**	**−0.336**	**−0.185**		
Fantasizing	Constant	15.494	1.099	13.337	17.652	0.066	13.97***
	**Analyzing**	**0.345**	**0.054**	**0.238**	**0.452**		df (3, 596)
	**Verbalizing**	**−0.114**	**0.036**	**−0.185**	**−0.043**		
	Identifying	−0.011	0.046	−0.100	0.079		
IRI Fantasy	Constant	68.590	2.613	63.459	73.721	0.229	35.18***
	Analyzing	−0.039	0.112	−0.258	0.180		df (5, 594)
	**Emotionalizing**	**−0.484**	**0.087**	**−0.654**	**−0.314**		
	**Fantasizing**	**−0.701**	**0.073**	**−0.844**	**−0.558**		
	**Verbalizing**	**−0.146**	**0.065**	**−0.274**	**−0.018**		
	Identifying	0.120	0.084	−0.045	0.286		

Subscales of the Bermond Vorst Alexithymia Scale: Emotionalizing, Analyzing, Verbalizing, Identifying, and Fantasizing. IRI Fantasy, Fantasy subscale from the Interpersonal Reactivity Index. LLCI and ULCI, lower limit and upper limit of the 95% confidence interval. Bolded coefficients are significant.

***p<0.001.

### 3.3 Discussion

Consistent with the results of Study 1, the link between EOT and IRI Fantasy scores was mediated by traits associated with SPS (here, fantasy proneness and emotional reactivity). In particular, we showed that those who have a stronger internal focus (low Analyzing) reported greater fantasy proneness and heightened emotional reactivity, and that both of these variables predicted higher scores on the IRI Fantasy subscale. We speculate that these results are largely driven by the fact that fantasy proneness and experiencing heightened sensitivity to subtle stimuli (including internally generated mental images) are SPS positive traits. We acknowledge, however, that emotional reactivity (as measured by the Emotionalizing subscale) also likely captures SPS negative traits to some degree. We return to this point below.

Mediation via Fantasizing makes sense if highly sensitive individuals who routinely daydream or fantasize in daily life also come to rely primarily on imagery-based strategies to reason and problem solve. In short, they may be more likely to become “visualizers” as opposed to “verbalizers” (Kozhevnikov et al., 2005). This may be why experiencing problems putting one’s feelings into words (high Verbalizing) predicted stronger fantasy proneness (low Fantasizing) in our model. By turning attention inward, strong “visualizers” would be able to maintain images of specific scenarios in an active state in working memory, where they could be combined with information stored in long-term memory to gain a deeper appreciation of the characters’ experiences. The idea that IRI Fantasy scores reflect this type of deep processing (rather than just frequent fantasizing) is consistent with the fact that scores on the IRI Fantasy subscale have been found to correlate with absorption (Wickramasekera and Szlyk, 2003). In other words, they correlate with the tendency to fully engage one’s attention in sensory and imaginative experiences in ways that alter one’s perception, memory, and mood in measurable ways. As suggested by the results of Study 1, this tendency would seem to fall primarily under the SPS positive trait cluster.

Mediation via Emotionalizing is consistent with Davis’s (1983) observation that scores on the IRI Fantasy scale correlate with scores on Mehrabian and Epstein’s (1972) Emotional Empathy Scale, which assesses characteristics such as extreme emotional responsiveness and susceptibility to emotional contagion. McQuarrie et al. (2023) have found such responses to be predicted by scores on both the OS (which loads on the positive SPS factor) and the HSPS (which is more heavily weighted to SPS negative traits), suggesting that high scores on Emotionalizing reflect stronger positive and negative SPS traits. This conclusion gains additional support from the fact that being more reactive (low Emotionalizing) was associated not only with feeling greater concern for others (Empathic Concern) but also greater uneasiness when witnessing others’ suffering (Personal Distress). In contrast to Emotionalizing, high scores on Verbalizing were weakly associated with lower scores on the IRI Fantasy, Empathic Concern, and Perspective Taking subscales, and with higher scores on Personal Distress. This latter observation supports the view that difficulties with emotional appraisal may contribute to problems empathizing with and acting compassionately toward others. Together, these results highlight the importance of considering the relative strength of specific traits linked to alexithymia and SPS when attempting to predict individual differences in a range of empathy-related constructs.

Although (as suggested by Study 1) SPS positive traits may prove to be the stronger mediator of the link between EOT and IRI Fantasy scores overall, we would remind the reader that the group scoring highest on the IRI Fantasy subscale in Study 1 was the group who scored high on both SPS positive and SPS negative traits (quadrant I in Figure 2A). We suspect that a closer examination of individuals’ reactions to specific scenarios might reveal that these individuals would be able to vividly imagine subtle features of the setting or the characters’ reactions. As a result, they might also experience even greater distress or unease than those who only score high on SPS negative traits when processing scenes that have negative valence (e.g., horror or true crime stories). Tentative support for this prediction comes from the facts that people who are good at mental imagery are not only more sensitive to incoming stimuli (Dance et al., 2021), but also exhibit larger fear responses (as indexed by changes in skin conductance) when reading scary stories, compared to those with limited imagery abilities (Wicken et al., 2021).

Jakobson and Rigby (2021) had previously argued that alexithymic individuals with strong EOT and poor fantasizing, and alexithymic individuals with weaker EOT and strong fantasizing, bear some similarities to the Type I and Type II alexithymia subtypes described by Bermond and colleagues (Bermond et al., 2006; Moormann et al., 2008), respectively. However, it is important to emphasize several key differences. Most importantly, Jakobson and Rigby (2021) found that, rather than showing *low* levels of emotional reactivity (a feature of Type I alexithymia), the group with strong EOT and an impoverished fantasy life tended to score moderately high on the HSPS (which, as noted earlier, is heavily weighted to SPS negative traits). Their responses on the Adolescent/Adult Sensory Profile (Brown and Dunn, 2002) indicated a sensory profile marked by sensitivity to and avoidance of unpleasant stimulation and by a strong tendency to avoid seeking out pleasurable stimulation—characteristics that could contribute to social withdrawal and put them at risk for depression. This group, then, has an alexithymia profile that most closely resembles that described by Preece et al. (2020): one marked by (relatively) high levels of emotional reactivity that disproportionately impacts the processing of negative emotions. In contrast, alexithymic individuals with weaker EOT and stronger fantasizing were sensitive to even subtle internally- and externally-generated stimuli, and were at even higher risk for both depression and anxiety. Based on the present results, we would predict that these individuals might be prone to experiencing intrusive emotional imagery and that they might ruminate on things that have gone wrong when imagining past events or worry excessively about what might happen when imagining the future.

## 4 General discussion

The results of the two studies reported here expand our understanding of the nature of the overlap between SPS and alexithymia and inform future research exploring the clinical implications of this overlap. In a recent report, Keefer et al. (2019) stated that, although their data supported the view that alexithymic traits are continuously distributed in the population, they “did not rule out the possibility that alexithymia itself may interact with other basic dimensions of personality—such as emotionalizing—to produce qualitatively distinct clinical profiles…[and] that emotionalizing, rather than any particular facet of alexithymia, is the likely linchpin of [Bermond et al.’s (2006)] proposed typology” (p. 371). The findings from a recent subtyping report (Jakobson and Rigby, 2021) and from the present investigation suggest instead that, although negative traits associated with SPS (i.e., heightened reactivity to unpleasant stimuli and situations) is seen in many people with alexithymia (as argued by Preece et al., 2017), it is individual differences in EOT, sensitivity to subtle stimuli, and fantasizing that best distinguish alexithymia subtypes. The fact that there may be two clinically-relevant subtypes of alexithymia that can be distinguished, in part, on the basis of these variables may explain why fantasizing deficits appear to be an inconsistent feature of alexithymia (Preece et al., 2020).

Other authors have reported negative associations between EOT and fantasizing (e.g., Bagby et al., 1994; Tibon et al., 2005; Henry et al., 2006; Taylor and Bagby, 2013), but this is the first study to show that this relationship is mediated by traits linked to SPS. We suggest that turning one’s attention inward could increase the salience of subtle, internally-generated stimuli like mental images (increasing the frequency with which they are recalled) and amplify one’s emotional reactions to imagined events, and that both of these effects might contribute, over time, to the development of a cognitive style characterized by a strong preference to apply imagery-based strategies for reasoning and problem solving. In contrast, we suspect that those who are more externally oriented may rely more on verbal strategies, leading the more dysregulated amongst them to, as Sifneos (1973) observed, “describe endlessly circumstances surrounding an event rather than the feelings [it engenders]” (p. 257). On this point, it is worth noting that the patients featured in Nemiah and Sifneos’ (1970) original report suffered from psychosomatic illnesses. We speculate that these patients, who the authors described as exhibiting problems with emotional appraisal and being externally oriented, likely scored higher on negative than positive SPS traits, overall.

## 5 Limitations and future directions

A limitation of Study 2 is that we did not assess SPS directly using established tools (e.g., the HSPS and the OS). As such, we could not determine the degree to which the Emotionalizing and Fantasizing subscales of the BVAQ correlate with these measures; this should be done in a future study. Nonetheless, a careful item analysis confirmed that low scores on these two BVAQ subscales do capture key features of SPS that have been described in the literature. This suggests that these two subscales have face validity as measures of SPS traits.

A second limitation of the current research is that we relied exclusively on self-report measures. Future researchers should attempt to obtain both subjective and objective measures of traits associated with alexithymia and SPS. It will be particularly important to conduct more research into the genetic, physiological, and neural bases of these partially heritable traits, and how they interact with environmental factors such as adverse life experiences. Existing self-report measures of alexithymia and SPS should be expanded to capture not only fantasy proneness but variables such as imagery vividness and cognitive style. Alternatively, including the IRI Fantasy subscale or the Object-Spatial Imagery and Verbal Questionnaire (Blazhenkova and Kozhevnikov, 2008) in one’s research protocol might be useful. We feel that gathering this additional information will prove important not only for theory building but also for the development of targeted treatment protocols for individuals suffering from mental health disorders. One could envisage, for example, that an alexithymic client who is a strong visualizer might respond differently than one who is not to training in self-regulatory strategies such as distancing (i.e., imagining that a distressing event happened to someone else or at a different time) or to imagery-based extinction procedures aimed at minimizing phobic responses.

It will be important in the future to conduct studies aimed at exploring the emotional lives of those who report a relatively strong external focus but few problems with emotional appraisal, and low levels of both positive and negative traits linked to SPS (i.e., who fall in quadrant III of Figure 2A). Jakobson and Rigby (2021) suggested that external events may be unlikely to trigger strong emotions in such individuals; as a result, they might show reduced emotional contagion and be less empathetic. In support of this, our current findings suggest that individuals with this profile find it difficult to imagine what characters in narratives might be thinking or feeling. Empathic deficits could increase their risk for certain conditions, such as antisocial personality disorder, clinically significant narcissism, or the grandiose form of subclinical narcissism.

## 6 Conclusion

Links between alexithymia and SPS have now been highlighted by several groups (e.g., Liss et al., 2008; Rigby et al., 2020; Attary and Ghazizadeh, 2021; Jakobson and Rigby, 2021; McQuarrie et al., 2023). Continuing to explore these links may help to explain some discrepancies in earlier work, offer new insights into individual differences in important cognitive processes such as imagery, and open new avenues of investigation that help refine theories about how emotions are generated, experienced, interpreted, and regulated. It is hoped that this work will also aid in the development of more effective, individualized treatments for mental health problems. The current study contributes to these efforts by offering a possible explanation for mixed findings in past research regarding fantasizing deficits in alexithymia.

## Data availability statement

The datasets presented in this study can be found in online repositories. The names of the repository/repositories and accession number(s) can be found below: “Individual differences in fantasy” repository at https://doi.org/10.34990/FK2/EPDHNN.

## Ethics statement

The studies involving humans were approved by Research Ethics Board 1 (Study 1) and Psychology/Sociology Research Ethics Board (Study 2) at the University of Manitoba. The studies were conducted in accordance with the local legislation and institutional requirements. The participants provided their written informed consent to participate in this study.

## Author contributions

LJ: Conceptualization, Formal analysis, Methodology, Supervision, Writing – original draft. AM: Conceptualization, Investigation, Methodology, Visualization, Writing – review & editing. CL: Conceptualization, Investigation, Methodology, Writing – review & editing. SS: Conceptualization, Methodology, Supervision, Writing – review & editing.
