# Supplementary material for: Traits linked to sensory processing sensitivity mediate the relationship between externally oriented thinking and fantasizing
Source: Front Psychol. 2024 Mar 12;15:1354120. doi: 10.3389/fpsyg.2024.1354120 (PMC10963545; doi:10.3389/fpsyg.2024.1354120)
Supplement: Supplementary file 1 [file Data_Sheet_1.PDF]

SUPPLEMENTARY MATERIALS

Table S1

Descriptive Statistics for Variables in Females and Males in Study 1

Variable	Females			Males		
	n	*M*	*SD*	*n*	*M*	*SD*
DIF	497	15.3	4.9	200	14.7	4.7
DDF	497	18.3	6.4	200	16.5	6.2
EOT	497	19.2	4.2	200	19.8	4.2
SPS pos	497	4.8	0.8	200	4.6	0.7
SPS neg	497	4.2	1.1	200	3.5	0.9
EC	385	3.0	0.7	111	2.6	0.7
FS	385	2.6	0.8	111	2.5	0.7
PD	385	1.9	0.7	111	1.5	0.7
PT	385	2.5	0.7	111	2.5	0.7

Note: Subscales of the Toronto Alexithymia Scale (TAS-20): DDF = difficulty describing feelings, DIF = difficulty identifying feelings, EOT = externally oriented thinking. SPS pos and SPS neg are the positive and negative composite scores for traits associated with sensory processing sensitivity. Subscales of the Interpersonal Reactivity Index (IRI): EC = Empathic concern, FS = Fantasy, PD = Personal Distress, PT = Perspective Taking.

Table S2

Zero-order Correlations Between Variables in Females and Males in Study 1

	2-tailed Pearson Correlations[a]								
	DDF	DIF	EOT	SPS pos	SPS neg	EC	FS	PD	PT
DDF	--	.644	.304	-.026	.354	-.076	-.238	.445	-.178
DIF	.674	--	.205	.154	.512	.009	-.064	.477	-.091
EOT	.279	.199	--	-.464	.031	-.207	-.292	.381	-.306
SPS pos	-.002	.096	-.427	--	.276	.211	.293	-.119	.448
SPS neg	.199	.334	-.047	.404	--	.218	.043	.531	.100
EC	-.096	-.004	-.247	.385	.234	--	.334	.166	.402
FS	.014	.061	-.199	.429	.266	.331	--	-.064	.271
PD	.296	.375	.174	.080	.474	.196	.095	--	-.125
PT	-.133	-.131	-.322	.352	.136	.444	.159	.010	--

Note: Correlations for males and females appear above and below the diagonal, respectively. Subscales of the Toronto Alexithymia Scale (TAS-20): DDF = difficulty describing feelings, DIF = difficulty identifying feelings, EOT = externally oriented thinking. SPS pos and SPS neg are the positive and negative composite scores for traits associated with sensory processing sensitivity. Subscales of the Interpersonal Reactivity Index (IRI): EC = Empathic concern, FS = Fantasy, PD = Personal Distress, PT = Perspective Taking.

[a] Values shown in gold, orange, and red represent small, medium, and large effect sizes, respectively. The df for the TAS-20 and HSPS measures are 200 for males and 497 for females. The df for the IRI subscales are 111 for males and 385 for females.

Table S3

Descriptive Statistics for Variables in Females and Males in Study 2

	Females (*n* = 382)		Males (*n* = 199)	
Variable	*M*	*SD*	*M*	*SD*
BVAQ_V	24.4	7.8	24.2	6.7
BVAQ_I	20.5	5.8	19.0	5.3
BVAQ_A	18.1	4.9	19.1	5.0
BVAQ_E	19.0	5.1	23.9	4.8
BVAQ_F	18.7	6.1	19.2	5.6
EC[a]	52.5	9.5	46.5	9.1
FS[a]	45.0	11.8	40.7	10.7
PD[a]	36.5	9.9	31.2	8.0
PT[a]	45.7	10.3	44.9	9.5

Note: Subscales of the Bermond-Vorst Alexithymia Questionnaire (BVAQ): V = Verbalizing, I = Identifying, A = Analyzing, E = Emotionalizing, F = Fantasizing. Subscales of the Interpersonal Reactivity Index (IRI): EC = Empathic concern, FS = Fantasy, PD = Personal Distress, PT = Perspective Taking.

[a] Items on the IRI were rated on a Likert scale ranging from 1 to 10, rather than the usual 1 to 5.

Table S4

Zero-order Correlations Between Variables in Females and Males in Study 2

	2-tailed Pearson Correlations[a]								
	BVAQ V	BVAQ I	BVAQ A	BVAQ E	BVAQ F	EC	FS	PD	PT
BVAQ V	--	.279	.420	.038	-.001	-.185	-.123	.164	-.132
BVAQ I	.413	--	.401	-.132	.202	-.001	-.033	.236	-.181
BVAQ A	.462	.341	--	.293	.233	-.215	-.259	.048	-.241
BVAQ E	.102	-.042	.474	--	.046	-.367	-.242	-.392	-.042
BVAQ F	-.006	-.009	.221	.220	--	-.078	-.343	-.022	-.184
EC	-.061	-.067	-.208	-.360	-.032	--	.276	.231	.335
FS	-.098	.009	-.189	-.274	-.425	.265	--	.139	.181
PD	.097	.239	-.030	-.379	-.090	.186	.204	--	-.054
PT	-.109	-.145	-.209	-.039	-.075	.445	.130	-.125	--

Note: Correlations for males and females appear above and below the diagonal, respectively. Subscales of the Bermond-Vorst Alexithymia Questionnaire (BVAQ): V = Verbalizing, I = Identifying, A = Analyzing, E = Emotionalizing, F = Fantasizing. Subscales of the Interpersonal Reactivity Index (IRI): EC = Empathic concern, FS = Fantasy, PD = Personal Distress, PT = Perspective Taking.

[a] Values shown in gold and orange represent small and medium effect sizes, respectively. The df for the BVAQ and IRI subscales are 199 for males and 382 for females.
